# Supplementary material for: Lactation Remodels the Maternal Lung to Promote Preemptive Innate Defense
Source: bioRxiv. 2026 Oct 8:2026.10.02.756233. Preprint. [Version 1] doi: 10.64898/2026.10.02.756233 (PMC13692044; doi:10.64898/2026.10.02.756233)
Supplement: Supplementary file 1 [file NIHPP2026.10.02.756233V1-supplement-1.pdf]

bioRxiv preprint doi: https://doi.org/10.64898/2026.10.02.756233; this version posted October 8, 2026. The copyright holder for this preprint (which was not certified by peer review) is the author/funder, who has granted bioRxiv a license to display the preprint in perpetuity. It is made available under a CC-BY 4.0 International license.

Supplementary Figures

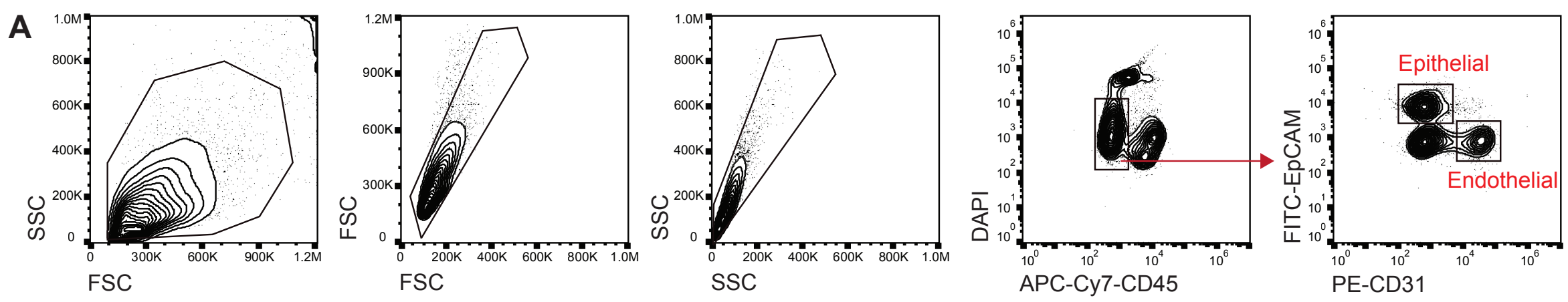

Cafiero *et al*. Figure S1

Figure S1. Gating strategy for lung epithelial and endothelial cell sorting. (**A**) Representative flow cytometry gating strategy used to sort lung epithelial and endothelial cells.

bioRxiv preprint doi: https://doi.org/10.64898/2026.10.02.756233; this version posted October 8, 2026. The copyright holder for this preprint (which was not certified by peer review) is the author/funder, who has granted bioRxiv a license to display the preprint in perpetuity. It is made available under a CC-BY 4.0 International license.

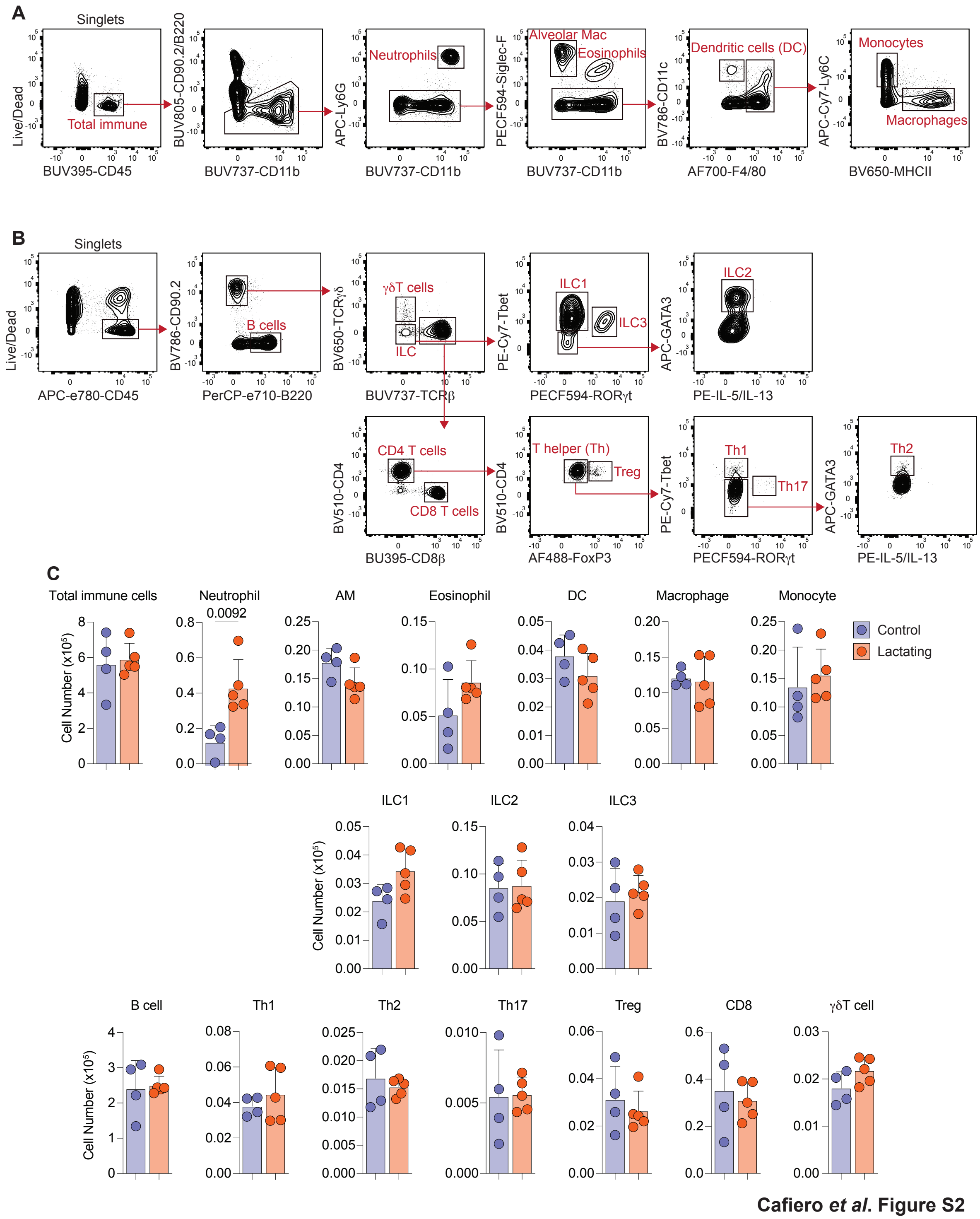

Figure S2. Lactating mice selectively increases neutrophils in the lung. (**A**) Flow cytometry gating scheme for myeloid cells. (**B**) Flow cytometry gating scheme for lymphoid cells. (**C**) Absolute cell number of myeloid and lymphoid cells in the lung of nulliparous control and lactating mice. Data in (C) represents mean ± SD and each dot represents one mouse. Data in (C) were analyzed by unpaired t-test. *P* values are indicated.

bioRxiv preprint doi: https://doi.org/10.64898/2026.10.02.756233; this version posted October 8, 2026. The copyright holder for this preprint (which was not certified by peer review) is the author/funder, who has granted bioRxiv a license to display the preprint in perpetuity. It is made available under a CC-BY 4.0 International license.

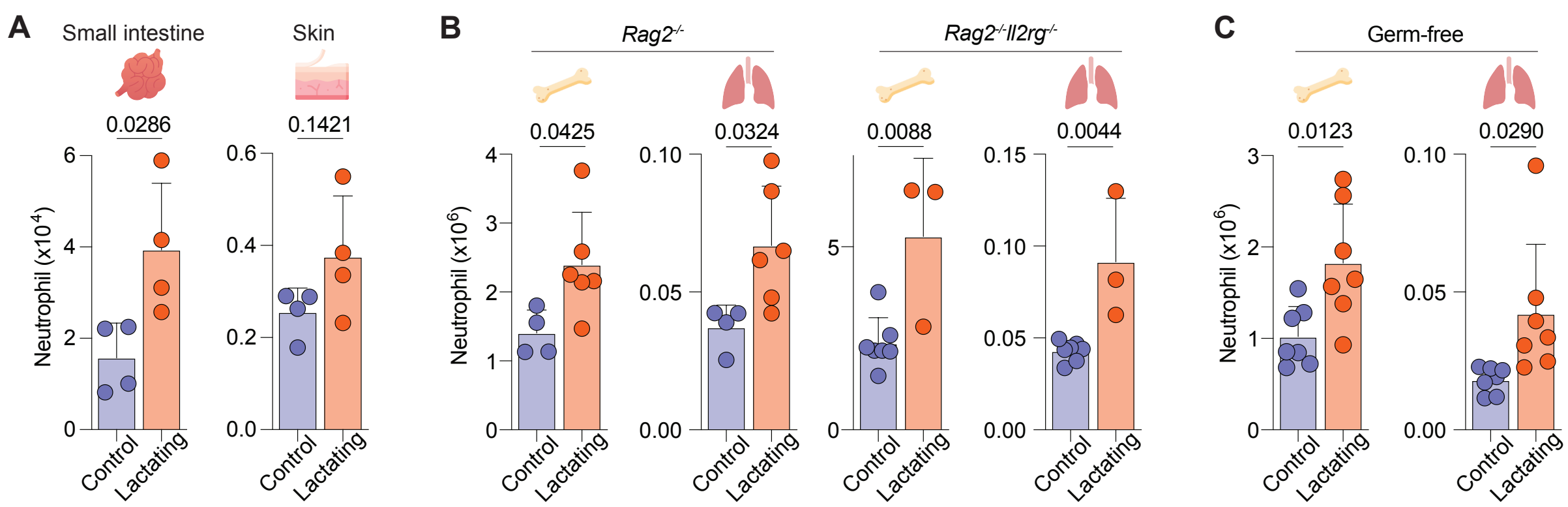

Cafiero *et al*. Figure S3

Figure S3. Lactation-associated granulocyte adaptations across tissues and experimental models. (**A**) Absolute cell number of neutrophils in small intestine and skin of nulliparous control and lactating mice. (**B**) Absolute cell number of neutrophils in bone marrow and lung of nulliparous control and lactating *Rag2*$^{-/-}$ (left) and *Rag2*$^{-/-}$*IL2rg*$^{-/-}$ (right) mice. (**C**) Absolute cell number of neutrophils in bone marrow and lung of germ-free nulliparous control and lactating mice. Data in (A), (B), and (C) represent mean ± SD and were analyzed by unpaired t-test. Each dot represents one animal. Data are representative of at least two experiments. *P* values are indicated.

bioRxiv preprint doi: https://doi.org/10.64898/2026.10.02.756233; this version posted October 8, 2026. The copyright holder for this preprint (which was not certified by peer review) is the author/funder, who has granted bioRxiv a license to display the preprint in perpetuity. It is made available under a CC-BY 4.0 International license.

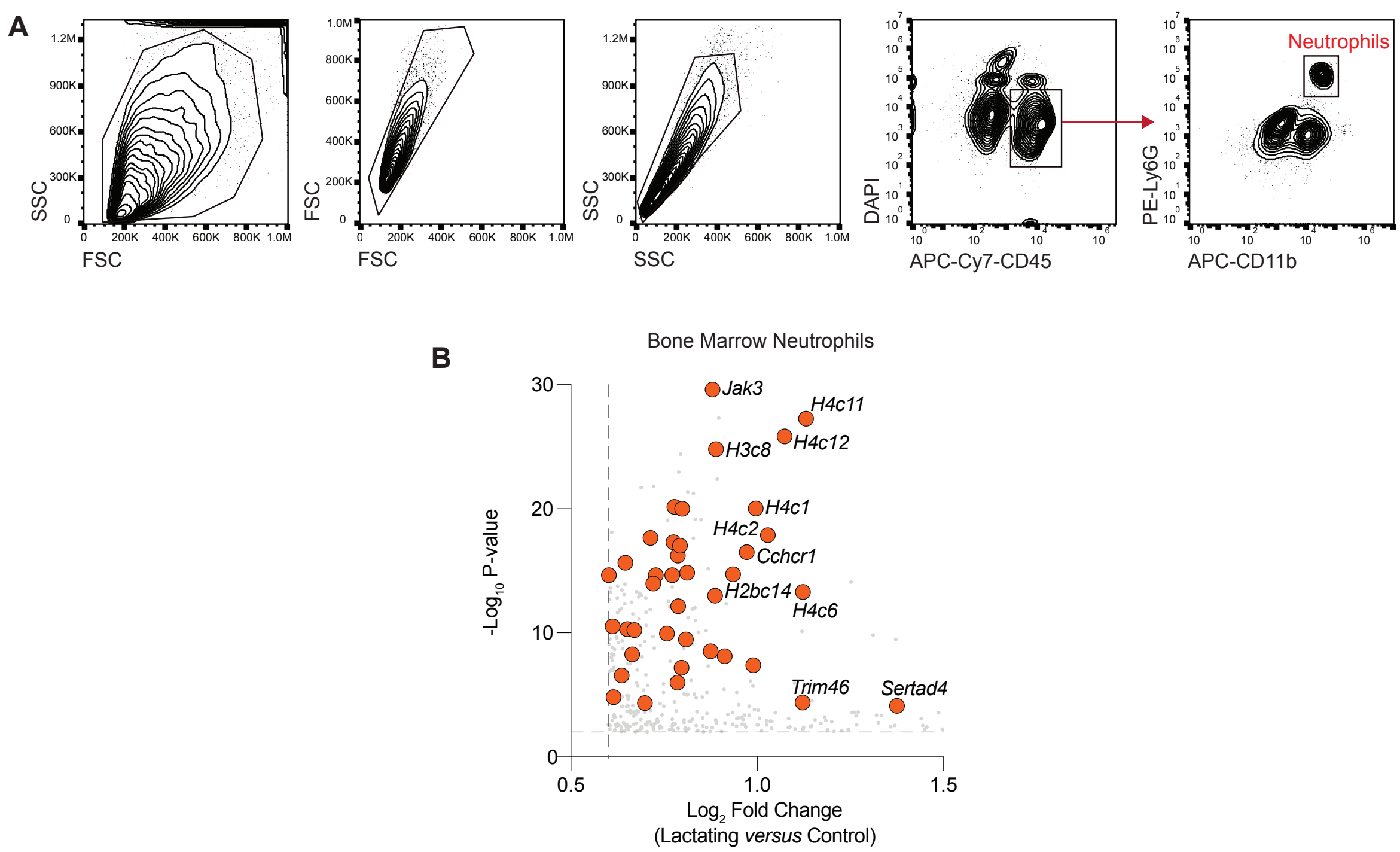

Cafiero *et al*. Figure S4

Figure S4. Neutrophils acquire activated signature following bone marrow egress. (**A**) Gating scheme of lung and bone marrow neutrophils. (**B**) Volcano plot of differentially overexpressed genes in bone marrow neutrophils from lactating versus nulliparous mice.

bioRxiv preprint doi: https://doi.org/10.64898/2026.10.02.756233; this version posted October 8, 2026. The copyright holder for this preprint (which was not certified by peer review) is the author/funder, who has granted bioRxiv a license to display the preprint in perpetuity. It is made available under a CC-BY 4.0 International license.

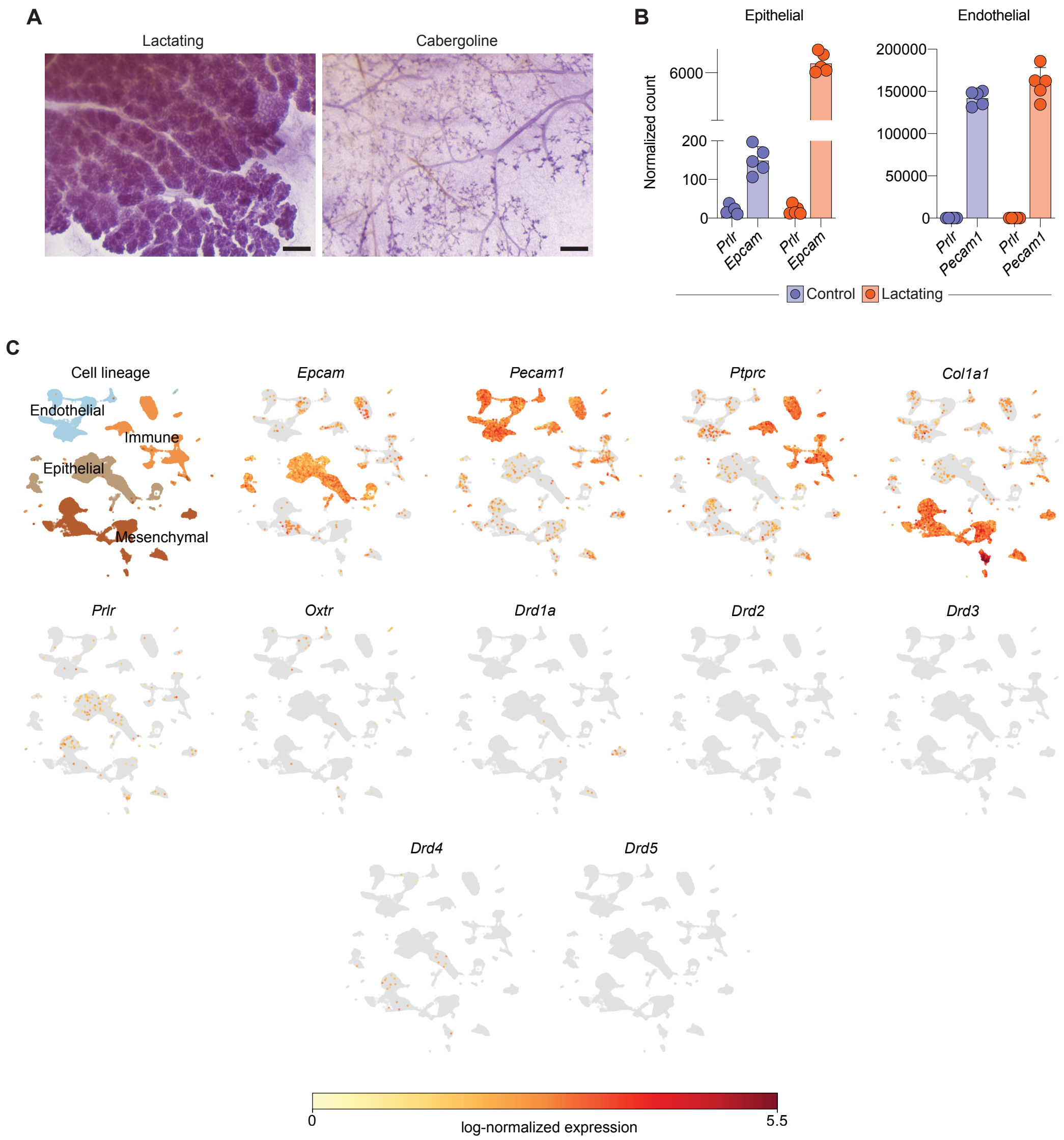

Cafiero *et al*. Figure S5

Figure S5. Prolactin, oxytocin and dopamine receptors show limited expression in the mouse lung. (**A**) Representative micrographs of mammary gland in lactating, and cabergoline treated lactating mice. Scale bars, 20 µm. (**B**) Normalized gene count of *Prlr* in lung epithelial (left) and endothelial (right) cells. **(C)** UMAP colored by log-normalized expression of broad cell-type marker genes: *Epcam* (epithelial), *Pecam1* (endothelial), *Ptprc* (immune), and *Col1a1* (mesenchymal), and prolactin receptor (*Prlr*), oxytocin receptor (*Oxtr*), and dopamine receptor (*Drd1a*, *Drd2*, *Drd3*, *Drd4*, and *Drd5*). Cells with zero detected counts are shown in grey. Color scale is shared across UMAPs and represents log-normalized expression clipped at the 99th percentile of expressing cells.

bioRxiv preprint doi: https://doi.org/10.64898/2026.10.02.756233; this version posted October 8, 2026. The copyright holder for this preprint (which was not certified by peer review) is the author/funder, who has granted bioRxiv a license to display the preprint in perpetuity. It is made available under a CC-BY 4.0 International license.

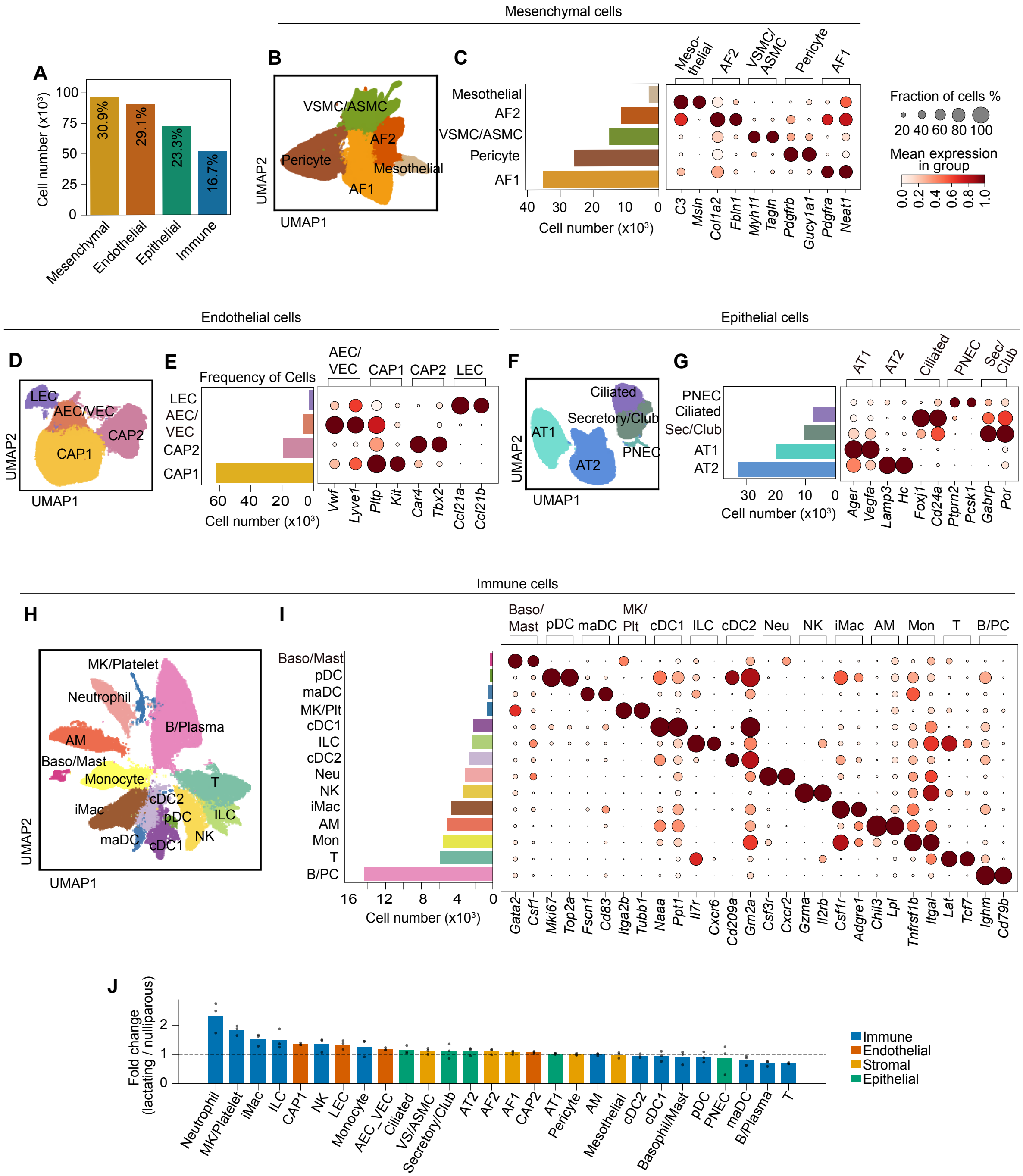

Cafiero *et al*. Figure S6

Figure S6. Spatial transcriptomic analysis of pulmonary remodeling. (**A**) Bar chart showing the percentage of cells per broad cell-type population. (**B**) UMAP representation of mesenchymal cells after independent reclustering, colored by subcluster identity. (**C**) Bar chart showing the number of cells per mesenchymal subcluster (left) and dot plot showing mean scaled expression of the top two marker genes per subcluster (right). Dot size indicates the fraction of cells

bioRxiv preprint doi: https://doi.org/10.64898/2026.10.02.756233; this version posted October 8, 2026. The copyright holder for this preprint (which was not certified by peer review) is the author/funder, who has granted bioRxiv a license to display the preprint in perpetuity. It is made available under a CC-BY 4.0 International license.

expressing each gene; color indicates mean scaled expression. (**D**) UMAP representation of endothelial cells after independent reclustering, colored by subcluster identity. (**E**) As in (**C**), for endothelial subclusters. (**F**) UMAP representation of epithelial cells after independent reclustering, colored by subcluster identity. (**G**) As in (**C**), for epithelial subclusters. (**H**) UMAP representation of immune cells after independent reclustering, colored by subcluster identity. (**I**) As in (**C**), for immune subclusters. Neu, neutrophil; B/PC, B cell/plasma cell; Mon, monocyte; MK/Plt, megakaryocyte/platelet. (**J**) Bar chart showing fold change in cell density (cells per μm^2; lactating versus nulliparous) for all clusters, colored by broad cell-type population. Dashed line indicates fold change = 1. Individual data points represent the fold change of each lactating replicate relative to mean nulliparous density.

bioRxiv preprint doi: https://doi.org/10.64898/2026.10.02.756233; this version posted October 8, 2026. The copyright holder for this preprint (which was not certified by peer review) is the author/funder, who has granted bioRxiv a license to display the preprint in perpetuity. It is made available under a CC-BY 4.0 International license.

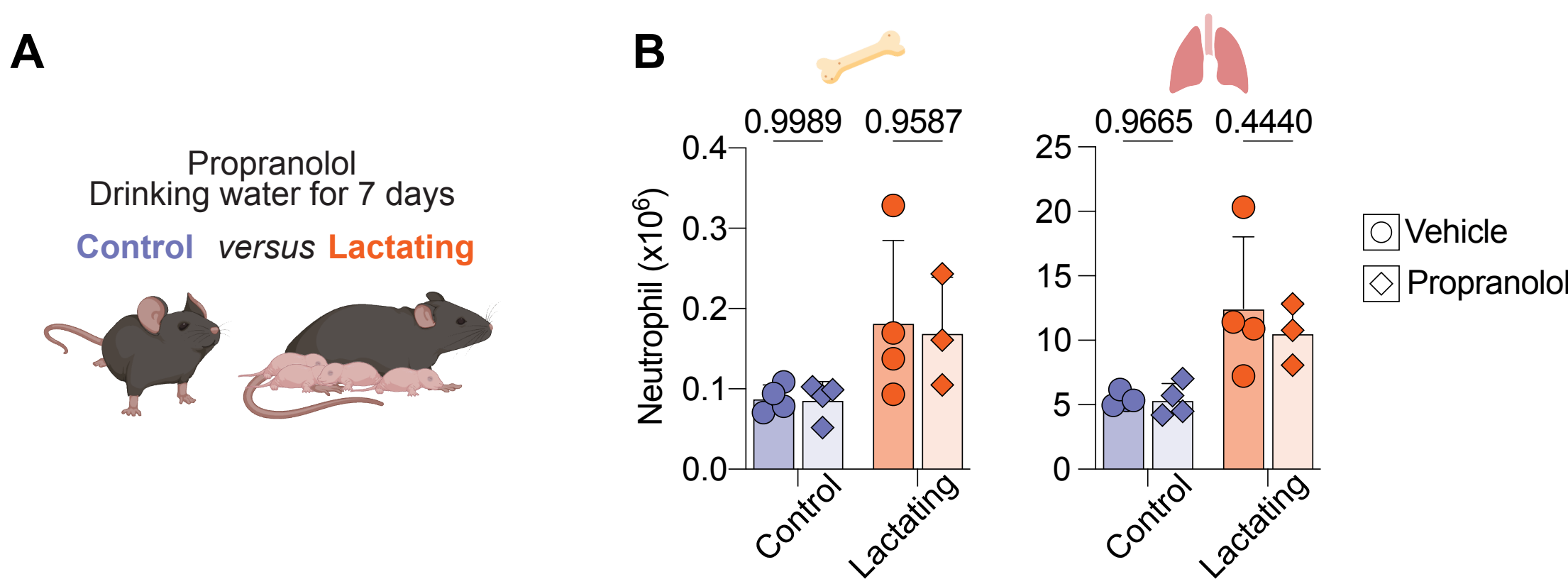

Cafiero *et al*. Figure S7

Figure S7 Limited contribution of β-adrenergic signaling to neutrophil accumulation. (**A**) Schematic of propranolol drinking water treatment. (**B**) Absolute cell number of bone marrow (left) and lung (right) neutrophils in vehicle and propranolol-treated control and lactating mice. Data in (B) represents mean ± SD and were analyzed by two-way ANOVA with Sidak's multiple comparisons correction. Each dot represents one animal, and *P* values are indicated.
